# Biological Activities of *Toninia candida* and *Usnea barbata* Together with Their Norstictic Acid and Usnic Acid Constituents

**DOI:** 10.3390/ijms131114707

**Published:** 2012-11-12

**Authors:** Branislav Ranković, Marijana Kosanić, Tatjana Stanojković, Perica Vasiljević, Nedeljko Manojlović

**Affiliations:** 1 Department of Biology, Faculty of Science, University of Kragujevac, 34000 Kragujevac, Serbia; E-Mail: rankovic@kg.ac.rs; 2 Institute of Oncology and Radiology of Serbia, 11000 Belgrade, Serbia; E-Mail: stanojkovict@dls.ncrc.ac.rs; 3 Department of Biology and Ecology, Faculty of Sciences and Mathematics, University of Niš, 18000 Niš, Serbia; E-Mail: perica@pmf.ni.ac.rs; 4 Department of Pharmacy, Faculty of Medical Sciences, University of Kragujevac, 34000 Kragujevac, Serbia; E-Mail: ntm@kg.ac.rs

**Keywords:** lichens, HPLC-UV, chemical composition, biological activities

## Abstract

The aim of this study was to investigate the chemical composition of acetone extracts of the lichens *Toninia candida* and *Usnea barbata* and *in vitro* antioxidant, antimicrobial, and anticancer activities of these extracts together with some of their major metabolites. The chemical composition of *T. candida* and *U. barbata* extracts was determined using HPLC-UV analysis. The major phenolic compounds in these extracts were norstictic acid (*T. candida*) and usnic acid (*U. barbata*). Antioxidant activity was evaluated by free radical scavenging, superoxide anion radical scavenging, reducing power and determination of total phenolic compounds. Results of the study proved that norstictic acid had the largest antioxidant activity. The total content of phenols in the extracts was determined as the pyrocatechol equivalent. The antimicrobial activity was estimated by determination of the minimal inhibitory concentration using the broth microdilution method. The most active was usnic acid with minimum inhibitory concentration values ranging from 0.0008 to 0.5 mg/mL. Anticancer activity was tested against FemX (human melanoma) and LS174 (human colon carcinoma) cell lines using the microculture tetrazolium test. Usnic acid was found to have the strongest anticancer activity towards both cell lines with IC_50_ values of 12.72 and 15.66 μg/mL.

## 1. Introduction

Lichens are symbiotic organisms built from fungi and a photosynthetic partner, which is either alga or Cyanobacterium [1–3]. They usually grow on rocks, non-fertile ground, and as epiphytes on trees and leaves [4]. For hundreds of years, lichens have been used in many Europeans country as a cure for stomach diseases, diabetes, coughs, pulmonary tuberculosis, wound curing, and dermatological diseases [5]. Besides, many sorts are used for human nutrition, animal nutrition, the production of colors, perfumes, alcohols and in the medical industry [6].

Lichens synthesize, mostly by a fungal metabolism, various bioactive secondary components, that sometimes constitute even more than 30% of the dry mass of talus [7]. They are found as crystal deposits on the surface of hiphes. They are poorly soluble in water and can usually be isolated from a lichen by means of organic diluents [8]. More than one hundred secondary metabolites, mainly monoaromatics, depsides, depsidones, pulvinates, dibenzofurans, anthraquinones and xanthones, characteristic of lichens have been detected and isolated [9]. Chemicals structures of these classes of compounds are similar and identification is often very difficult.

Lichen metabolites exert a large variety of biological actions including antibiotic, antimycotic, antiviral, anti-inflammatory, analgesic, antipyretic, antiproliferative and cytotoxic effects [10,11]. Here, we report the chemical composition of *Toninia candida* and *Usnea barbata* extracts using HPLC-UV analysis and *in vitro* antioxidant, antimicrobial and anticancer activities of these lichens and their norstictic acid and usnic acid constituents.

## 2. Results and Discussion

**Figure 1:**
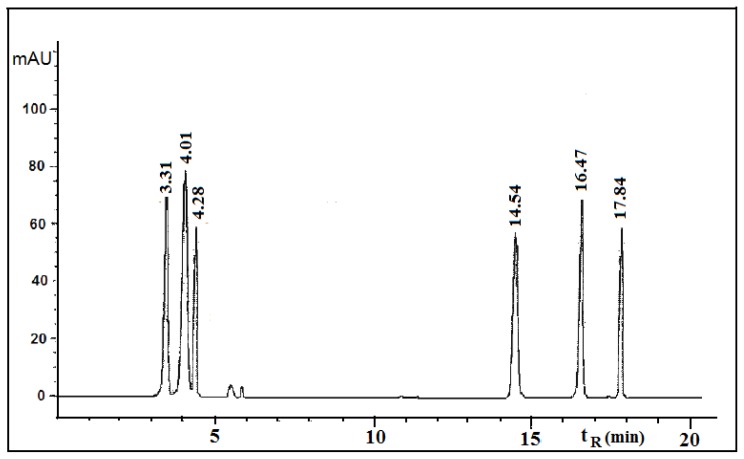
Chromatogram of the standards used for identification of the compounds present in *Toninia candida* and *Usnea barbata*.

**Figure 2:**
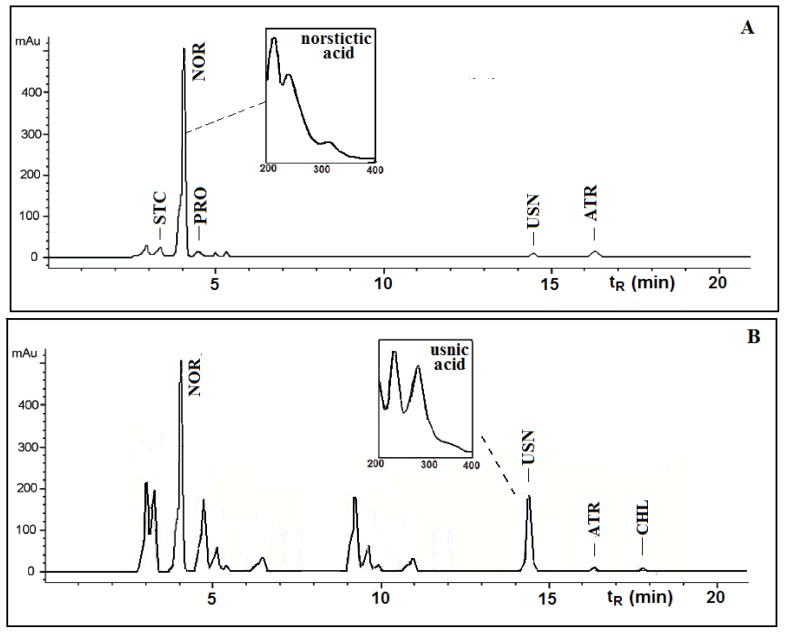
HPLC chromatograms acquired at 254 nm of the acetone extracts of *Toninia candida* (**A**) and *Usnea barbata* (**B**). Chromatographic peak identities are reported in Table 1.

**Figure 3:**
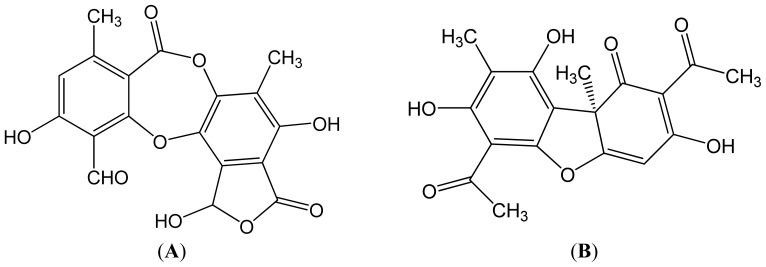
Structures of the identified compounds. (**A**) Norstictic acid; (**B**) Usnic acid.

This paper deals with the phytochemical analysis of acetone extracts from the species *T. candida* and *U. barbata*, lichens growing in Serbia, and the isolation of their major metabolites. Figures 1 and 2 show the HPLC chromatograms of the standards and the acetone extracts from the species *T. candida* and *U. barbata*. In addition to the main chemical compound (norstictic acid), stictic acid, protocetraric acid, usnic acid and atranorin were also identified in the acetone extract of *T. candida*. On the other hand, the acetone extract of *U. barbata* showed the presence of norstictic acid, usnic acid, atranorin and chloroatranorin. Identification of these compounds was achieved by comparison of their *t*_R_ values with the standard substances previously isolated from lichens. Norstictic acid has specific UV spectra different from other depsidones with absorption maxima at 212, 238 and 310 nm. Compared with many other lichen substances, the UV spectrum of usnic acid is very specific and this acid can easily be identified by its UV spectrum (absorbance maxima at 234 and 284 nm) and its retention time by HPLC. After HPLC analysis of the tested lichen extracts, their major lichen metabolites, norstictic and usnic acids, were isolated from the acetone extracts of *T. candida* and *U. barbata,* respectively. Table 1 shows the retention time of the detected lichen substances and their absorbance maxima (nm). Norstictic acid belongs to the class of depsidones, while usnic acid is a well-known lichen metabolite with a dibenzofurane structure. The structures of the detected compounds are shown in Figure 3. These pure compounds were further used for antioxidant, antimicrobial and anticancer investigations.

The scavenging DPPH radicals of the studied samples are shown in Table 2. The IC_50_ values of all extracts and compounds ranged from 102.65 to 979.30 μg/mL. There was a statistically significant difference between samples and the control (*p* < 0.05). Among the tested extracts, the acetone extract from *T. candida* showed the largest DPPH radical scavenging activity (IC_50_ = 115.77 μg/mL). The lichen components isolated demonstrated very strong DPPH radical scavenging activity, larger than those from extracts. Usnic acid showed a smaller DPPH radical scavenging activity than norstictic acid. The IC_50_ values for norstictic acid and usnic acid were 102.65 and 130.73 μg/mL respectively.

The results of the reducing power assay of the tested extracts and compounds are summarized in Table 3. Higher absorbance indicates higher reducing power. Among the lichen extracts, *T. candida* showed the highest reducing power. Norstictic acid and usnic acid showed a very high reducing power, which was higher than those from extracts.

The scavenging of superoxide anion radicals by the tested lichen extracts and compounds is shown in Table 2. There was a statistically significant difference between samples and the control (*p* < 0.05). The lichen components isolated showed the highest superoxide anion radical scavenging activity (the IC_50_ was 133.46 μg/mL for norstictic and 197.28 μg/mL for usnic acid). The scavenging activities of tested extracts were somewhat lower.

The total phenolic content of the tested lichen extracts are given in Table 4. The total amount of phenolic compounds was determined as the pyrocatechol equivalent using an equation obtained from a standard pyrocatechol graph. The total phenolic contents of the acetone extracts of *T. candida* and *U. barbata* were 49.81 and 31.25 μg PE/mg respectively.

Various antioxidant activities were compared to ascorbic acid. The results showed that the standard antioxidant had a stronger activity than the tested samples.

The tested lichen extracts have a strong antioxidant activity against various oxidative systems *in vitro*. The strong antioxidant activity of the tested lichen extracts is correlated with a high content of total phenols. In fact, it was observed that the examined lichen extracts with the higher content of phenols exert a stronger radical scavenging effect, suggesting that phenolics are the main agents for their antioxidant activity. These results mostly correspond to the literature, where we have found a number of reports for the antioxidant activity of extracts with a high content of phenolic compounds [12]. In most lichens, phenols, including depsides, depsidones, and dibenzofurans, are important antioxidants because of their ability to scavenge free radicals such as singlet oxygen, superoxide, and hydroxyl radicals [10]. As well, in our experiment the isolated components norstictic acid and usnic acid which are classified as phenols, also exhibited a significantly stronger antioxidant activity than the extracts, which indicates the important role of phenols in antioxidant activity. However, some authors believe that the antioxidant activity of extracts may not necessarily be correlated with the content of polyphenolics, suggesting that the antioxidant activity of different lichens may also depend on other, non-phenol components [13].

The antioxidant effect of some other lichens was also studied by other researchers. For example, Luo *et al.*[14] determined the antioxidant activity of methanol extracts from the lichen *Thamnolia vermicularis*. Praveen Kumar *et al.*[15] determined the antioxidant activity of the extracts of the lichen *Ramalina hossei* and *Ramalina conduplicans.* Manojlović *et al.*[16] explored the antioxidant properties of *Laurera benguelensis*.

The antimicrobial activities of the lichen extracts and lichen components against the test microorganisms are shown in Table 5. The extract from *U. barbata* showed a moderate antibacterial and antifungal activity. It inhibited the microorganisms tested at concentrations from 0.125 to 12.5 mg/mL. The extract from *T. candida* inhibited all the tested microorganisms, but at higher concentrations. The lichen components isolated demonstrated very strong antimicrobial activity. The MIC for different components relative to the tested microorganisms ranged from 0.0008 to 1 mg/mL. The strongest antimicrobial activity was found in usnic acid, which in extremely low amounts (Table 5) inhibited all species of bacteria and fungi.

The antimicrobial activity was compared with the standard antibiotics, streptomycin (for bacteria) and ketoconazole (for fungi). The results showed that standard antibiotics had a similar or stronger activity than the tested samples as shown in Table 5. In a negative control, DMSO had no inhibitory effect on the tested organisms.

In our experiments, the tested lichen extracts showed a relatively strong antimicrobial activity but the antimicrobial activity of their components was much stronger. This means that the lichen components are responsible for the antimicrobial activity of the lichens. Differences in antimicrobial activity of different species of lichens are probably a consequence of the presence of different components with varying antimicrobial activity [17]. However, it is necessary to understand that extracts are mixtures of natural compounds, and their antimicrobial activity is not only a result of the different activities of the individual components but may also be the result of their interactions, which can cause different effects on the overall activity of the extracts.

The intensity of the antimicrobial effect depended on the species of organism tested. The extracts and compounds used in this study, had stronger antibacterial than antifungal activity. This observation is in accordance with other studies [17,18], focused on antimicrobial activity which have demonstrated that bacteria are more sensitive to the antimicrobial activity than the fungi due to differences in the composition and permeability of the cell wall. The cell wall of Gram-positive bacteria is composed of peptidoglucanes and teichoic acids, while the cell wall of Gram-negative bacteria is composed of peptidoglucanes, lipopolysacharides and lipoproteins [10,19]. The cell wall of fungi is poorly permeable and consists of polysaccharides such as hitchin and glucan [20].

Numerous lichens were screened for antimicrobial activity in search of new antimicrobial agents. Kosanić *et al.*[10] find antimicrobial activity for the acetone extract of the lichens *Umbilicaria crustulosa*, *U. cylindrica*, and *U. polyphylla*. Similar results were reported by Karthikai Devi *et al.*[21] for different extracts extracted from the lichen *Roccella belangeriana*. Goel *et al.*[22] found that the lichen *Parmotrema reticulatum* had a strong antimicrobial influence.

The cytotoxic activity of the tested lichen extracts and compounds against the tested cell lines is shown in Table 6. The tested samples exhibited high cytotoxic activity against the target cells *in vitro*. The IC_50_ value for different samples relative to the tested cells ranged from 12.72 to 56.96 μg/mL. The best cytotoxic activity was exhibited for usnic acid. The IC_50_ against FemX and LS174 cell lines was very low (12.72 and 15.66 μg/mL, respectively).

As shown in the table, the positive control (Cis-DDP) had a slightly better cytotoxic activity than the tested samples.

The effect of tested samples on the cell cycle was evaluated using flow cytometric analysis. Table 7 shows a cell-cycle distribution of FemX and LS174 cells incubated in the absence or presence of compounds (IC_50_) for 24 h, approximately double the time of this cell line. Treatment of FemX cells with tested extracts for 24 h led to a significant decrease of G1 cells and an increase of G2/M cells, revealing cell cycle arrest at this phase. On the other hand, treatment of the tested compounds led to an increase in the number of cells in the sub-G1 phase whiled the percentage of cells in the S-phase and G2/M phase remained unchanged compared to the controls. Interestingly, LS174 cells treated with the tested samples showed a significant increase of the sub-G1 phase and concomitant decrease in G2/M was observed, supporting a G1 phase arrest. These results suggested that the tested samples have a prominent ability to induce apoptosis in FemX and LS174 cells.

In the present study, the results clearly demonstrate that the studied lichen components induced a significant cytotoxic effect on the tested cancer cell lines, which was stronger than the lichen extracts. Some literature data have reported that lichen components are responsible for the anticancer activities of lichens [23,24]. However, it is difficult to determine the contribution of individual components on the overall anticancer effect. Often, the activity of the extracts may be the result of synergistic or antagonistic effects of several compounds.

The importance of lichens as anticancer agents has been confirmed in recent years, suggesting that lichens can be used as biological agents in the treatment of cancer. The mechanism of action of the tested extracts and their compounds is yet to be tested. Further research will be necessary for fractionation in order to identify the compounds responsible for the observed anti-tumor effects, and to establish the opportunities for reinforcement activities as well as to improve the selectivity.

Until now, only a few research examples have proved that lichens have anticancer activity. Bezivin *et al.*[25] reported a significant anticancer effect for *Parmelia caperata*, *Cladonia convoluta*, *Cladonia rangiformis*, *Platismatia glauca* and *Ramalina cuspidata*. Manojlović, *et al.*[11] explored the anticancer properties of *Thamnolia vermicularis*. Trigiani *et al.*[26] found strong anticancer activity for *Xanthoria parietina.*

## 3. Experimental Section

### 3.1. Lichen Samples

Lichen samples of *Toninia candida* (Weber) Th. Fr., and *Usnea barbata* (L.) Mott., were collected from Kopaonik, Serbia, in September of 2011. The demonstration samples are preserved in facilities of the Department of Biology and Ecology of Kragujevac, Faculty of Science. Determination of the investigated lichens was accomplished using standard methods.

### 3.2. Preparation of the Lichen Extracts

Finely dry ground thalli of the examined lichens (100 g) were extracted using acetone in a Soxhlet extractor. The extracts were filtered and then concentrated under reduced pressure in a rotary evaporator. The dry extracts were stored at −18 °C until they were used in the tests. The extracts were dissolved in 5% dimethyl sulphoxide (DMSO) for the experiments. DMSO was dissolved in sterile distilled water to the desired concentration.

### 3.3. High Performance Liquid Chromatography (HPLC) Analysis

The acetone extracts of the tested lichens were redissolved in 500 μL of acetone and analyzed on an Agilent Technologies HPLC instrument 1200 Series with C18 column (25 cm × 4.6 mm, 10 μm) and a UV spectrophotometric detector with detection at 254, 280, and 320 nm. The same compounds were detected at all selected wavelengths. Methanol-water-phosphoric acid (80:20:0.9, *v*/*v*/*v*) was used as a solvent. Phosphoric acid was analytical-grade reagent. Methanol was of HPLC grade and was purchased from Merck (Darmstadt, Germany). Deionized water used throughout the experiments was generated by a Milli-Q academic water purification system (Milford, MA, USA). The sample injection volume was 10 μL. The flow rate was 1.0 mL/min. The standards used were obtained from the following sources: norstictic acid (*t*_R_ = 4.01 ± 0.20 min) was isolated from the lichen *Ramalina furinacea*, stictic acid (*t*_R_ = 3.31 ± 0.01 min) from the lichen *Xanthoparmelia conspersa*, protocetraric acid (*t*_R_ = 4.28 ± 0.05 min) from the lichen *Cetraria islandica*, usnic acid (*t*_R_ = 14.54 ± 0.30 min), atranorin (*t*_R_ = 16.47 ± 0.30 min) and chloroatranorin (*t*_R_ = 17.84 min) from the lichen *Evernia prunastri*. The standard samples were isolated in our laboratory and their structures were confirmed by melting point, elemental analysis and ^1^H and ^13^C-NMR data [27].

### 3.4. The Isolation of Norstictic Acid from the Lichen Toninia candida

The dried acetone extract of the lichen *T. candida* (500 mg) was dissolved in benzene. After filtration, the residue was fractioned on a silica gel 60 column (under 0.063 mm; 230 mesh). The column was eluted with methanol-chloroform gradient solvent (10:1 and 5:1) yielding seven fractions. The fifth eluted fraction of the lichen extract contained norstictic acid (176 mg). This compound was used for structure identification and determination of the antioxidant, antimicrobial and cytotoxic activities. For experiments, norstictic acid was dissolved in 5% DMSO.

### 3.5. The Isolation of Usnic Acid from the Lichen Usnea barbata

The dried acetone extract of the lichen *U. barbata* (500 mg) was dissolved in benzene. The precipitate which formed on cooling was collected and analyzed by HPLC. HPLC analysis showed that the precipitate contained, besides usnic acid, a small amount of atranorin and chloroatranorine. Therefore, the precipitate was fractionated on a silica gel 60 column (under 0.063 mm; 230 mesh) using cyclohexane-ethyl acetate (75:25, *v*/*v*). The first eluted compound was usnic acid, which was further recrystallized from chloroform-ethanol, yielding 95 mg of a pure yellow compound. This compound was further purified by co-chromatography and used for structure identification and determination of antioxidant, antimicrobial and cytotoxic activities. For experiments, usnic acid was dissolved in 5% (DMSO). Since it is well known that usnic acid has strong antioxidant, antimicrobial, cytotoxic *etc.* activities, it was used in this study for comparison with the examined activities of other tested samples.

Norstictic acid and usnic acid were identified by their melting point, elemental analysis and ^1^H and ^13^C-NMR data [27]. The purity of the isolated compounds was determined by HPLC-DAD and amounted to 98.6% and 97.2%, for usnic acid and norstictic acid, respectively.

### 3.6. Antioxidant Activity

#### 3.6.1. Scavenging DPPH Radicals

The free radical scavenging activity of samples was measured by 1,1-diphenyl-2-picryl-hydrazil (DPPH). The method used is similar to the method previously used by some authors [28,29], but was modified in some of the details. Two milliliters of a methanol solution of the DPPH radical at a concentration of 0.05 mg/mL with 1 mL of test sample (1000, 500, 250, 125 and 62.5 μg/mL) was placed in a cuvette. The mixture was shaken vigorously and allowed to stand at room temperature for 30 min. After that, the absorbance was measured at 517 nm in spectrophotometer (“Jenway” UK). Ascorbic acid was used as the positive control. The DPPH radical concentration was calculated using the following equation:

(1) $$\text{DPPH scavenging effect }(\%)=[(A_{0}-A_{1})/A_{0}]\times 100$$

where *A*_0_ is the absorbance of the negative control and *A*_1_ is the absorbance of reaction mixture or standard.

The inhibition concentration at 50% inhibition (IC_50_) was the parameter used to compare the radical scavenging activity.

#### 3.6.2. Reducing Power

The reducing power of samples was determined according to the Oyaizu method [30]. One milliliter of test sample (1000, 500, 250, 125 and 62.5 μg/mL) was mixed with 2.5 mL of phosphate buffer (2.5 mL, 0.2 M, pH 6.6) and potassium ferricyanide (2.5 mL, 1%). The mixtures were incubated at 50 °C for 20 min. Then, trichloroacetic acid (10%, 2.5 mL) was added to the mixture and centrifuged. Finally, the upper layer was mixed with distilled water (2.5 mL) and ferric chloride (0.5 mL; 0.1%). The absorbance of the solution was measured at 700 nm in spectrophotometer (“Jenway” UK). Higher absorbance of the reaction mixture indicated that the reducing power had increased. Ascorbic acid was used as positive control.

#### 3.6.3. Superoxide Anion Radical Scavenging Activity

The superoxide anion radical scavenging activity of samples was detected according to the method of Nishimiki *et al.*[31]. Briefly, 0.1 mL of test sample (1000, 500, 250, 125 and 62.5 μg/mL) was mixed with 1 mL nitroblue tetrazolium (NBT) solution (156 μM in 0.1 M phosphate buffer, pH 7.4) and 1 mL nicotinamide adenine dinucleotide (NADH) solution (468 μM in 0.1 M phosphate buffer, pH 7.4). The reaction was started by adding 100 μL of phenazine methosulphate (PMS) solution (60 μM in 0.1 M phosphate buffer, pH 7.4). The mixture was incubated at room temperature for 5 min, and the absorbance was measured at 560 nm in spectrophotometer (“Jenway” UK) against blank samples. Decreased absorbance indicated increased superoxide anion radical scavenging activity. Ascorbic acid was used as positive control. The percentage inhibition of superoxide anion generation was calculated using the following formula:

(2) $$\text{Superoxide anion scavenging activity }(\%)=[(A_{0}-A_{1})/A_{0}]\times 100$$

where *A*_0_ is the absorbance of the negative control and *A*_1_ is the absorbance of reaction mixture or standards.

The inhibition concentration at 50% inhibition (IC_50_) was the parameter used to compare the radical scavenging activity.

#### 3.6.4. Determination of Total Phenolic Compounds

Total soluble phenolic compounds in the acetone extracts were determined with Folin-Ciocalteu reagent according to the method of Slinkard and Singleton [32] using pyrocatechol as a standard phenolic compound. Briefly, 1 mL of the extract (1 mg/mL) in a volumetric flask diluted with distilled water (46 mL) was taken and one milliliter of Folin-Ciocalteu reagent was added and the content of the flask mixed thoroughly. After 3 min, 3 mL of sodium carbonate (2%) was added and then was left to stand for 2 h with intermittent shaking. The absorbance was measured at 760 nm in spectrophotometer (“Jenway” UK). The total concentration of phenolic compounds in the extract was determined as micrograms of pyrocatechol equivalent (PE) per milligram of dry extract by using an equation that was obtained from a standard pyrocatechol graph as follows:

(3) $$\text{Absorbance}=0.0057\times \text{total phenols }[\mu \text{g PE}/\text{mg of dry extracts}]-0.1646\;(R^{2}=0.9203)$$

### 3.7. Antimicrobial Activity

#### 3.7.1. Microorganisms and Media

In this study, the following bacteria were used as test organisms: *Bacillus mycoides* (ATCC 6462), *Bacillus subtilis* (ATCC 6633), *Staphylococcus aureus* (ATCC 25923), *Escherichia coli* (ATCC 25922) and *Klebsiella pneumoniae* (ATCC 13883). All the bacteria used were obtained from the American Type Culture Collection (ATCC). Their identification was confirmed at the Microbiological Laboratory of Kragujevac, University of Kragujevac, Department of Biology. The fungi used as test organisms were: *Aspergillus flavus* (ATCC 9170), *Aspergillus fumigatus* (DBFS 310), *Candida albicans* (ATCC 10231), *Penicillium purpurescens* (DBFS 418) and *Penicillium verrucosum* (DBFS 262). They were from the from the American Type Culture Collection (ATCC) and the mycological collection maintained by the Mycological Laboratory within the Department of Biology of Kragujevac University’s Faculty of Science (DBFS). Bacterial cultures were maintained on Müller-Hinton agar substrates (Torlak, Belgrade). Fungal cultures were maintained on potato dextrose (PD) agar and Sabourad dextrose (SD) agar (Torlak, Belgrade). All cultures were stored at 4 °C and sub-cultured every 15 days.

The sensitivity of microorganisms to the tested samples was tested by determining the minimal inhibitory concentration (MIC).

Bacterial inoculi were obtained from bacterial cultures incubated for 24 h at 37 °C on Müller-Hinton agar substrate and brought up by dilution according to the 0.5 McFarland standard to approximately 10^8^ CFU/mL. Suspensions of fungal spores were prepared from fresh mature (3- to 7-day-old) cultures that grew at 30 °C on a PD agar substrate. Spores were rinsed with sterile distilled water, used to determine turbidity spectrophotometrically at 530 nm, and then further diluted to approximately 10^6^ CFU/mL according to the procedure recommended by NCCLS [33].

#### 3.7.2. Minimal Inhibitory Concentration (MIC)

The minimal inhibitory concentration (MIC) was determined by the by the broth microdilution method by using 96-well micro-titer plates [34]. A series of dilutions with concentrations ranging from 50 to 0.195 mg/mL for extracts and 4 to 0.0004 mg/mL for compounds was used in the experiment against every microorganism tested. The starting solutions of test samples were obtained by measuring off a certain quantity of extract and dissolving it in DMSO. Two-fold dilutions of test samples were prepared in Müller-Hinton broth for bacterial cultures and SD broth for fungal cultures. The minimal inhibitory concentration was determined with resazurin. Resazurin is an oxidation-reduction indicator used for the evaluation of microbial growth. It is a blue non-fluorescent dye which turns pink and fluorescent when reduced to resorufin by oxidoreductases within viable cells. The boundary dilution without any changing color of resazurin was defined as the minimal inhibitory concentration (MIC) for the tested microorganism at the given concentration. Streptomycin was used in the case of bacteria and ketoconazole in the case of fungi as a positive control growth inhibition. A DMSO solution was used as a negative control for the influence of the solvents. All experiments were performed in triplicate.

### 3.8. Cytotoxic Activity

#### 3.8.1. Cell Lines

The human melanoma FemX and human colon carcinoma LS174 cell lines were obtained from the American Type Culture Collection (Manassas, VA, USA). Both cancer cell lines were maintained in the recommended RPMI-1640 medium supplemented with 10% heat-inactivated (56 °C) fetal bovine serum, l-glutamine (3 mM), streptomycin (100 mg/mL), penicillin (100 IU/mL), and 25 mM HEPES and adjusted to pH 7.2 by bicarbonate solution. Cells were grown in a humidified atmosphere of 95% air and 5% CO_2_ at 37 °C.

#### 3.8.2. Treatment of Cell Lines

Stock solutions (100 mg/mL) of test samples, made in dimethylsulfoxide (DMSO), were dissolved in the corresponding medium to the required working concentrations. Neoplastic FemX cells (5000 cells per well) and neoplastic LS174 cells (7000 cells per well) were seeded into 96-well microtiter plates, and 24 h later, after the cell adherence, five different, double diluted, concentrations of the investigated compounds, were added to the wells. The final concentrations applied to target cells were 200, 100, 50, 25 and 12.5 μg/mL, except for the control wells, where only nutrient medium was added to the cells. The nutrient medium was RPMI 1640 medium, supplemented with l-glutamine (3 mM), streptomycin (100 mg/mL), penicillin (100 IU/mL), 10% heat inactivated (56 °C) fetal bovine serum (FBS) and 25 mM Hepes, and was adjusted to pH 7.2 by bicarbonate solution. The cultures were incubated for 72 h.

#### 3.8.3. Determination of Cell Survival (MTT test)

The effect of test samples on cancer cell survival was determined by MTT test (microculture tetrazolium test), according to Mosmann [35] with modification by Ohno and Abe [36], 72 h upon addition of the compounds, as described earlier. Briefly, 20 μL of MTT solution (5 mg/mL PBS) were added to each well. Samples were incubated for a further 4 h at 37 C in 5% CO_2_ and a humidified air atmosphere. Then, 100 μL of 10% SDS was added to the extract of the insoluble product formazan, resulting from the conversion of the MTT dye by viable cells. The number of viable cells in each well was proportional to the intensity of the light absorbance, which was then read in an ELISA plate reader at 570 nm. Absorbance (A) at 570 nm was measured 24 h later. To get the cell survival (%), A of a sample with cells grown in the presence of various concentrations of the investigated test samples it was divided by the control optical density (the A of control cells grown only in nutrient medium), and multiplied by 100. It was implied that A of the blank was always subtracted from A of the corresponding sample with target cells. IC_50_ concentration was defined as the concentration of an agent inhibiting cell survival by 50%, compared with a vehicle-treated control. As a positive control *cis*-diamminedichloroplatinum (*cis*-DDP) was used. All experiments were done in triplicate.

#### 3.8.4. Flow Cytometry Analysis

Cellular DNA content and cell distribution were quantified by flow cytometry using propidium iodide (PI). Cells (3 × 10^5^ cells/well) were seeded in 6-well plates and incubated with or without IC_50_ concentration of investigated compounds for 24 h. After treatment, the cells were collected by trypsinization, and fixed in ice-cold 70% ethanol at −20 °C overnight. After fixation, the cells were washed in PBS and pellets obtained by centrifugation were treated with RNase (100 μg/mL) at 37 °C for 30 min and then incubated with propidium iodide (PI) (40 μg/mL) for at least 30 min. DNA content and cell cycle distribution were analyzed using a Becton Dickinson FAC-Scan flow cytometer. Flow cytometry analysis was performed by using a CellQuestR (Becton Dickinson, San Jose, CA, USA), on a minimum of 10,000 cells per sample [37].

### 3.9. Statistical Analyses

Statistical analyses were provided by using EXCEL and SPSS software packages. To determine the statistical significance of antioxidant activity, the student’s *t*-test was used. All values are expressed as mean ± SD of three parallel measurements.

## 4. Conclusion

In conclusion, it can be stated that the tested lichen extracts and their compounds have a strong antioxidant, antimicrobial and anticancer activity *in vitro.* Based on these results, lichens appear to be good natural antioxidant, antimicrobial and anticancer agents and also, could be of significance in the food industry and in the control of various human, animal and plant diseases. Further studies should be carried out to search for new compounds from lichens that exhibit strong antioxidant, antimicrobial and anticancer activities.

## Figures and Tables

**Table 1:** Retention time of the examined lichen substances and their absorbance maxima (nm).

Compound	Substance Class	Retention time (*t*_R_ ± SD) * (min)	Absorbance maxima (nm) UV spectrum
Norstictic acid	Depsidone	4.01 ± 0.10	212, 239, 310 m
Usnic acid	Dibenzofurane	14.54 ± 0.20	234, 282

* Values are the means of three determinations ± SD;

m minor absorbance maximum.

**Table 2:** DPPH radical scavenging activity and superoxide anion scavenging activity of acetone extracts of *Toninia candida* and *Usnea barbata* and their compounds.

Lichen species and compounds	DPPH radical scavenging IC_50_ (μg/mL)	Superoxide anion scavenging IC_50_ (μg/mL)
*T. candida*	115.77	221.52
*U. barbata*	667.97	979.30
Norstictic acid	102.65	133.46
Usnic acid	130.73	197.28
Ascorbic acid	6.42	115.61

**Table 3:** Reducing power of acetone extracts of *Toninia candida* and *Usnea barbata* and their compounds.

Lichen species and compounds	Absorbance (700 nm)				

	1000 μg/mL	500 μg/mL	250 μg/mL	125 μg/mL	62.5 μg/mL
*T. candida*	0.0890	0.0555	0.0369	0.0299	0.0186
*U. barbata*	0.0324	0.0189	0.0153	0.0049	0.0035
Norstictic acid	0.8773	0.6641	0.0870	0.0422	0.0275
Usnic acid	0.6723	0.5468	0.0692	0.0249	0.0101
Ascorbic acid	2.113	1.654	0.0957	0.0478	0.0297

**Table 4:** Total phenolic contents of acetone extracts of *Toninia candida* and *Usnea barbata*.

Lichen species and compounds	Phenolic content (μg PE/mg of extract)
*T. candida*	49.81 ± 1.065
*U. barbata*	31.25 ± 1.013

**Table 5:** Antimicrobial activity of acetone extracts of *Toninia candida* and *Usnea barbata* and their compounds.

Microorganisms	*Toninia candida* MIC *	*Usnea barbata* MIC *	Norstictic Acid MIC *	Usnic acid MIC *	Antibiotics (S, K) MIC *
*B. mycoides*	3.12 *	0.25	0.25	0.0008	7.81
*B. subtilis*	3.12	0.25	0.25	0.0008	7.81
*E. coli*	6.25	0.50	0.50	0.25	31.25
*K. pneumoniae*	6.25	0.125	0.25	0.0625	1.95
*S. aureus*	12.5	0.50	0.25	0.125	31.25
*A. flavus*	50	12.5	0.50	0.50	3.9
*A. fumigatus*	25	12.5	0.50	0.25	3.9
*C. albicans*	12.5	6.25	0.50	0.125	1.95
*P. purpurescens*	25	12.5	1	0.50	3.9
*P. verrucosum*	25	12.5	1	0.50	3.9

* Minimum inhibitory concentration (MIC); Values given as mg/mL for tested samples and as μg/mL for antibiotics; Values are the mean of three replicate; Antibiotics: K, ketoconazole (for fungi); S, streptomycin (for bacteria).

**Table 6:** Growth inhibitory effects of acetone extracts of *Toninia candida* and *Usnea barbata* and their compounds on FemX and LS 174 cell lines expressed as IC_50_ values (μg/mL).

Lichen species and compounds	FemX IC_50_ (μg/mL)	LS 174 IC_50_ (μg/mL)
*T. candida*	55.09 ± 1.87	56.96 ± 0.87
*U. barbata*	24.81 ± 0.39	34.89 ± 1.54
Norstictic acid	20.74 ± 0.21	28.08 ± 1.38
Usnic acid	12.72 ± 0.35	15.66 ± 1.45
Cis-DDP	0.94 ± 0.35	2.3 ± 0.31

**Table 7:** Effect of *Toninia candida* and *Usnea barbata* and their compounds on cell cycle progression.

	Apoptotic cells sub-G1	G1	S	G2/M
**FemX**				
Control	0.30	58.19	15.42	22.87
*T. candida*	5.34	47.64	15.09	31.93
*U. barbata*	6.90	46.65	9.36	37.09
Norstictic acid	18.20	43.81	15.30	18.62
Usnic acid	10.18	54.38	14.64	20.37

**LS 174**				
Control	2.55	49.53	12.06	32.21
*T. candida*	15.83	47.35	15.89	20.66
*U. barbata*	13.87	46.47	16.82	22.85
Norstictic acid	16.92	52.34	15.28	16.49
Usnic acid	12.89	52.77	15.06	19.28

Effect of compounds on cell cycle phase distribution. FemX and LS 174 cell lines were exposed to compounds (IC_50_ μg/mL) for 24 h and then collected for analysis of cell cycle phase distribution using flow cytometry. Percentage of cells under different stages of cell cycles (sub-G1, G1, S, G2/M) is shown.
